# Magnifying Endoscopic Features of Follicular Lymphoma Involving the Stomach: A Report of Two Cases

**DOI:** 10.1155/2016/2082698

**Published:** 2016-09-22

**Authors:** Masaya Iwamuro, Katsuyoshi Takata, Seiji Kawano, Nobuharu Fujii, Yoshiro Kawahara, Tadashi Yoshino, Hiroyuki Okada

**Affiliations:** ^1^Department of Gastroenterology and Hepatology, Okayama University Graduate School of Medicine, Dentistry, and Pharmaceutical Sciences, Okayama 700-8558, Japan; ^2^Department of General Medicine, Okayama University Graduate School of Medicine, Dentistry, and Pharmaceutical Sciences, Okayama 700-8558, Japan; ^3^Department of Pathology, Okayama University Graduate School of Medicine, Dentistry, and Pharmaceutical Sciences, Okayama 700-8558, Japan; ^4^Department of Hematology and Oncology, Okayama University Hospital, Okayama 700-8558, Japan; ^5^Department of Endoscopy, Okayama University Hospital, Okayama 700-8558, Japan

## Abstract

A 70-year-old woman presented with follicular lymphoma involving the stomach, duodenum, jejunum, bone, and lymph nodes. Esophagogastroduodenoscopy revealed multiple depressed lesions in the stomach. Examination with magnifying endoscopy showed branched abnormal vessels along with gastric pits, which were irregularly shaped but were preserved. The second case was a 45-year-old man diagnosed with stage II_1_ follicular lymphoma with duodenal, ileal, and colorectal involvement, as well as lymphadenopathy of the mesenteric lymph nodes. Esophagogastroduodenoscopy performed six years after the diagnosis revealed multiple erosions in the gastric body and angle. Magnifying endoscopic observation with narrow-band imaging showed that the gastric pits were only partially preserved and were destroyed in most of the stomach. Branched abnormal vessels were also seen. Pathological features were consistent with follicular lymphoma in both cases. The structural differences reported between the two cases appear to reflect distinct pathologies. Disappearance of gastric pits in the latter case seems to result from loss of epithelial cells, probably due to chronic inflammation. In both cases, branched abnormal vasculature was observed. These two cases suggest that magnified observations of abnormal branched microvasculature may facilitate endoscopic detection and recognition of the extent of gastric involvement in patients with follicular lymphoma.

## 1. Introduction

Follicular lymphoma is the second most frequent subtype of lymphoid malignancies observed in western countries. In patients with follicular lymphoma, the gastrointestinal tract can be primarily or secondarily involved [1]. Most gastrointestinal involvement of follicular lymphoma is found in the small intestine, especially in the duodenum [2–4], whereas gastric involvement is less frequent. Therefore, the macroscopic and microscopic features of follicular lymphoma involving the stomach have not been fully revealed to date.

Recently we experienced two cases of systemic follicular lymphoma involving the stomach. This paper focuses on the pathologic and endoscopic features of gastric lesions of follicular lymphoma. We also speculate on the pathophysiological processes behind the microstructural findings in both presented cases.

## 2. Case Report

### 2.1. Case  1

**Figure 1:**
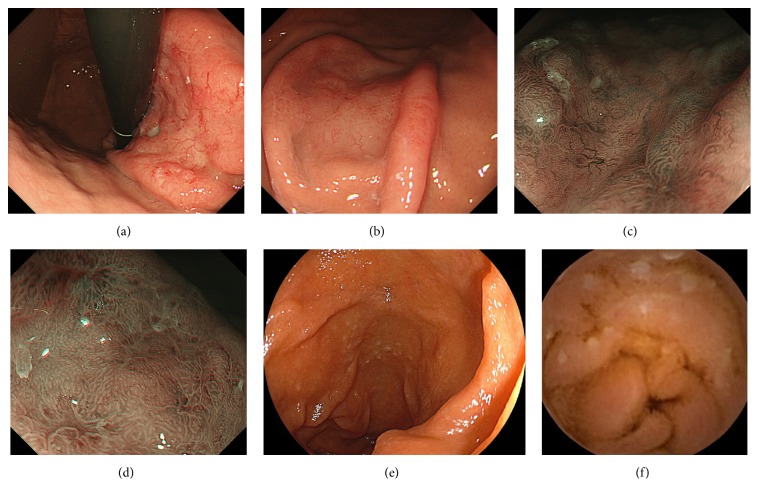
Endoscopic images from case 1. Slightly depressed lesions with thickened peripheral folds were seen in the upper (a) and lower gastric bodies (b). Branched abnormal vessels (c) and irregularly shaped gastric pits (c, d) were observed by using magnifying endoscopy with narrow-band imaging. In the duodenum, multiple whitish depositions were detected (e). Video capsule enteroscopy showed whitish granules in the jejunum (f).

**Figure 2:**
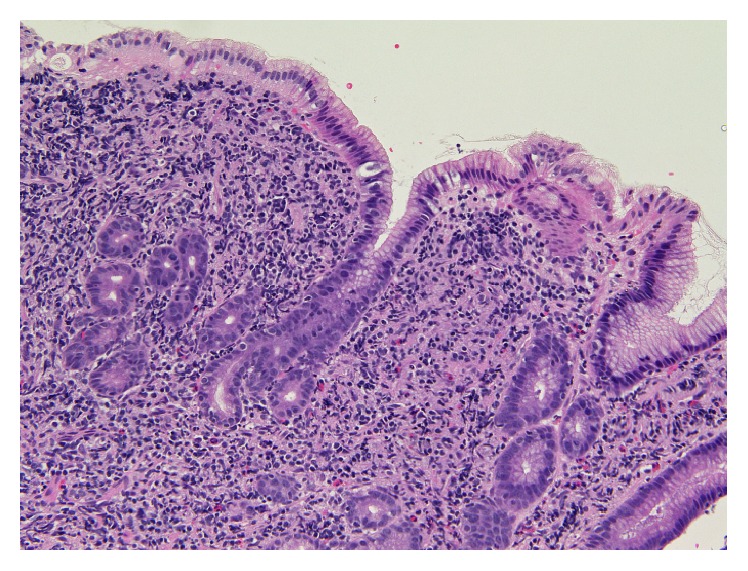
Histological image of stomach tissue biopsies from case 1. Dense, diffuse infiltration of small to medium-sized lymphoma cells was observed (hematoxylin and eosin staining, ×40). Lymphoepithelial lesions were absent.

A 70-year-old woman underwent a screening esophagogastroduodenoscopy at her family clinic during a routine medical checkup, and a slightly depressed area was found in the gastric corpus. The patient was referred to Okayama University Hospital for further investigation and treatment. Esophagogastroduodenoscopy (GIF-H260Z; Olympus, Tokyo) showed slightly depressed lesions with thickened peripheral folds in the upper (Figure 1(a)) and lower gastric body (Figure 1(b)). Magnifying endoscopic observation with narrow-band imaging revealed branched abnormal vessels (Figure 1(c)). The gastric pits in the lesions were found to be irregular (Figures 1(c) and 1(d)), and the appearance of the gastric pits was denser than that of the peripheral intact mucosa. No absence or destruction of gastric pits was observed. In addition, multiple whitish depositions were identified in the duodenum (Figure 1(e)). The patient had tested negative for* Helicobacter pylori* infection. Biopsy samples from the peripheral intact mucosa had no neoplastic cells, whereas samples from the gastric lesions showed dense, diffuse infiltration of small- to medium-sized lymphoma cells (Figure 2). Lymphoepithelial lesions were absent. Immunohistochemistry analysis showed that the lymphoma cells were positive for CD20, CD10, and BCL2, while they were negative for CD3. The antibodies used to analyze the case were as follows (clone, dilutions): CD20 (L26, 1 : 200), CD10 (56C6, 1 : 50), BCL2 (3.1, 1 : 200), and CD3 (PS-1, 1 : 50). Positivity was determined when 30% or more lymphoma cells were positive for their antibodies. Fluorescence in situ hybridization analysis revealed that translocation t(14;18)(q32;q21) was present in the gastric lymphoma cells. Pathological features were consistent with follicular lymphoma. Biopsy specimens obtained from the duodenum showed small- to medium-sized neoplastic lymphoid cells forming lymphoid structures. These pathological findings are representative of duodenal follicular lymphomas [3].

Video capsule enteroscopy showed whitish granules in the jejunum (Figure 1(f)). Computed tomography (CT) and positron emission tomography revealed involvement of multiple lymph nodes around the stomach, aorta, mesentery, and rectum. A bone marrow aspirate and biopsy revealed infiltration of the neoplastic cells into the bone marrow. Consequently, we diagnosed the case as systemic follicular lymphoma involving the stomach, duodenum, jejunum, intraabdominal lymph nodes, and bone marrow. The clinical stage was classified as stage IV.

### 2.2. Case  2

**Figure 3:**
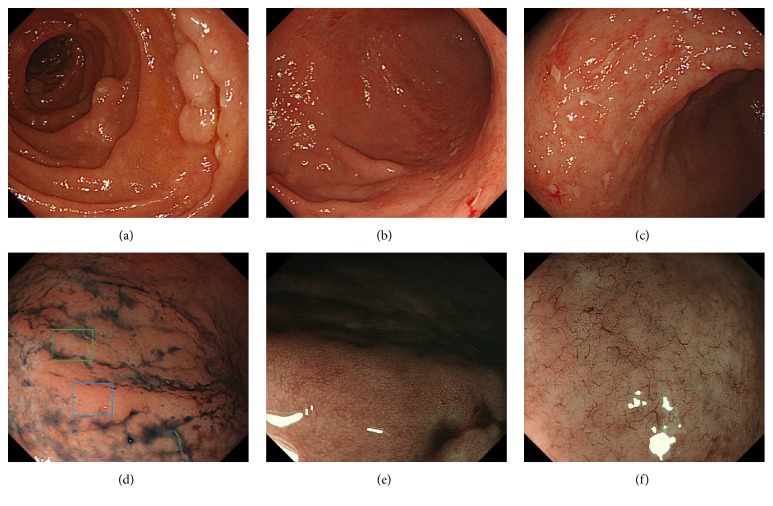
Endoscopic images from case 2. Esophagogastroduodenoscopy revealed multiple whitish depositions in the duodenum (a). In the stomach, multiple erosions in the body and angle were observed (b, c). There were no erosions in the greater curvature of the gastric body (d). Magnifying endoscopic observation with narrow-band imaging of the gastric body ((d), blue square) showed that the gastric pits were only partially preserved (e). Imaging a second area ((d), green square) showed destruction of gastric pits and branched abnormal vessels (f).

**Figure 4:**
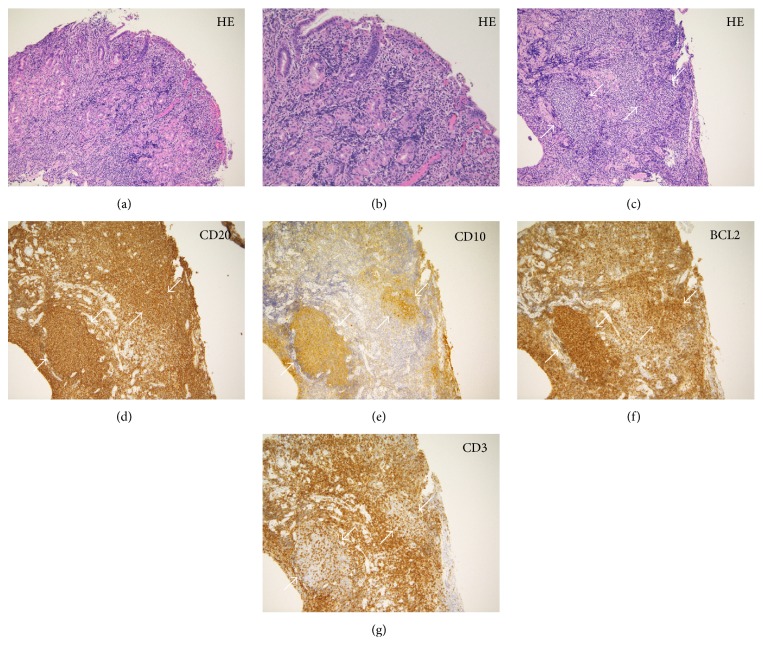
Histological images of stomach tissue biopsies from case 2. Dense, diffuse infiltration of small- to medium-sized lymphoma cells was observed, in addition to the existence of granulation tissues, with preserved epithelial cells, from biopsy samples taken from the gastric mucosa where intact pits were observed (hematoxylin and eosin staining, (a) ×20, (b) ×40). An image from biopsy samples taken from where damaged gastric pits were observed shows lymphoma infiltration, but with loss of epithelial cells ((c), hematoxylin and eosin staining, ×20). Neoplastic lymphoid follicles are shown (arrows). Lymphoma cells were positive for CD20 ((d), ×20), CD10 ((e), ×20), and BCL2 ((f), ×20) and negative for CD3 ((g), ×20).

**Figure 5:**
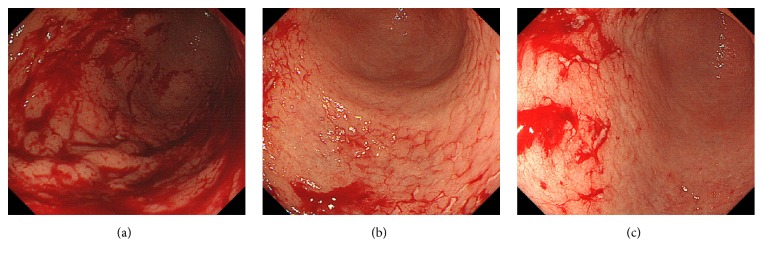
Esophagogastroduodenoscopy performed eight years after the initial diagnosis. Spontaneous bleeding from the gastric mucosa was observed.

A 45-year-old man was diagnosed with stage II_1_ follicular lymphoma with duodenal, ileal, colorectal involvement and lymphadenopathy of the mesenteric lymph nodes. Lymphoma progression with axillary and inguinal lymph node swelling was noted one year later, but the patient was kept under active surveillance (i.e., watch and wait). Two years after the initial diagnosis, gastric involvement was diagnosed. Esophagogastroduodenoscopy performed six years after the diagnosis revealed whitish depositions in the duodenum (Figure 3(a)). Multiple erosions in the gastric body and angle were also observed (Figures 3(b) and 3(c)). No erosions were observed in the greater curvature of the gastric body (Figure 3(d)). However, magnifying endoscopic observations using narrow-band imaging showed that the gastric pits were only partially preserved (Figure 3(e)), whereas they were heavily destroyed for the most part (Figure 3(f)). Branched abnormal vessels were also seen in areas where the gastric pits were absent.* Helicobacter pylori* titer was negative in this patient. Biopsy samples from the gastric lesions showed a diffuse infiltration of small- to medium-sized lymphoma cells, in addition to the existence of granulation tissue and lymphoid follicle (Figures 4(a), 4(b), and 4(c)). Immunohistochemistry confirmed the diagnosis of gastric follicular lymphoma, with staining positive for CD20 (Figure 4(d)), CD10 (Figure 4(e)), and BCL2 (Figure 4(f)). Staining for CD3 expression was negative (Figure 4(g)) in the neoplastic lymphoid cells. Pathological analysis found that epithelial cells were preserved in biopsy samples taken from the gastric mucosa where intact gastric pits were observed (Figures 4(a) and 4(b)). In contrast, epithelial cell loss was observed in biopsy samples taken from areas with damaged or absent gastric pits (Figure 4(c)). Esophagogastroduodenoscopy performed eight years after the initial diagnosis revealed an increased number of gastric erosions and spontaneous bleeding from the gastric mucosa (Figure 5). Because lymphoma progression was prominent in the stomach, rituximab monotherapy was initiated to prevent gastric bleeding.

## 3. Discussion

Lymphoma derived gastric lesions present with diverse endoscopic features, varying from mass-forming tumors to diffuse infiltrating lesions or superficial mucosal changes [5]. Moreover, formation of multiple lesions in the stomach is frequently observed in lymphomas [6]. Since most cases presenting with gastric lymphoma are extranodal marginal-zone lymphoma of mucosa-associated lymphoid tissue (MALT lymphoma) or diffuse large B-cell lymphoma [7], only a few articles describing gastric lesions from follicular lymphoma have been reported. In cases of gastrointestinal follicular lymphoma, involvement of the duodenum is a predominant feature [2, 8]. Recent reports have revealed a high proportion of cases ranging from 66.7% to 100%, with extensive involvement of the jejunum and/or ileum [3, 9–14]. Meanwhile, involvement of the stomach is infrequent in follicular lymphoma. A previous report by Takata et al. described observation of duodenal lesions in 111/125 cases (88.8%), jejunal lesions in 50/125 cases (40.0%), and ileal lesions in 28/125 cases (22.4%), while gastric lesions were found in only 2/125 cases (1.6%) [3]. Macroscopic features described for gastric lesions of follicular lymphoma include shallow depressed lesions [15], thickened rugae exhibiting a slight redness [16], elevated nodular lesions [17], mucosal inflammation and ulceration [18], multiple small ulcerations [19], and a submucosal tumor-like lesion [20, 21]. Based on the varied descriptions used to describe follicular lymphoma, no specific macroscopic features have been identified in association with the gastric lesions.

Although gastric involvement is infrequent in follicular lymphoma patients, it is important to evaluate the gastric mucosa during both the initial evaluation and the follow-up period as part of a thorough diagnostic examination. During the initial diagnostic workup of follicular lymphoma patients, determination of the extent of disease present is recommended for stage I and II patients who are being considered for radiotherapy as a curative treatment. Staging in these patients is important since disease relapse tends to occur outside the involved field of radiation [22]. Even though case  2 did not have any gastric involvement at the initial staging, it is possible for gastric lesions to emerge during the follow-up period. Case  2 required initiation of rituximab monotherapy to prevent gastric bleeding, since spontaneous bleeding from gastric mucosa occurred secondary to the gastric follicular lymphoma lesions.

To the best of our knowledge, magnifying endoscopic features observed in gastric involvement of follicular lymphoma have not been reported to date. As described above, involvement of the stomach is more frequent in MALT lymphoma, which has been a well-recognized entity among lymphomas involving the stomach since first described by Isaacson and Wright in 1983 [23]. Typical magnifying endoscopic features of MALT lymphoma include the appearance of abnormal vessels and the destruction of gastric pits [24, 25]. Ono et al. investigated 11 cases of gastric MALT lymphoma and using magnified endoscopy observed the two aforementioned features in all cases [24, 26]. Moreover, after achieving a complete response of MALT lymphoma, by* Helicobacter pylori* eradication, abnormal vessels were no longer detected and gastric pits reemerged, though those pits were irregularly patterned and had a different form and a different density from those in the adjacent intact mucosa. Consequently, unusual-shaped vasculature, a nonstructural pattern, and destruction of gastric pits appear to be specific for untreated gastric MALT lymphomas.

In both of the present cases, magnifying endoscopic observations with narrow-band imaging showed abnormal branched vessels. As described above, branched microvasculature observed in our cases was similar to the reported features in cases of MALT lymphoma [24]. Moreover, such branched vessels can be seen in* H. pylori*-associated chronic gastritis and diffuse type of gastric cancer [24, 27]. Therefore, distinguishing gastric follicular lymphoma from other lymphoma subtypes and other gastric diseases seems difficult or impossible by considering this feature alone. However, despite the limitations associated with magnified endoscopic observation, we consider that understanding magnifying endoscopic features of gastric follicular lymphoma lesions will aid endoscopists for the following reasons. First, magnifying endoscopy may aid in the determination of appropriate biopsy sites. In case 1, biopsy samples from the depressed lesion with abnormal branched vessels showed lymphoma cells, whereas samples from the peripheral mucosa had no neoplastic cells [26]. Second, detection of gastric involvement may alter treatment strategy in patients with follicular lymphoma. For example, rituximab monotherapy was initiated in case 2 to prevent gastric mucosa bleeding after esophagogastroduodenoscopy confirmed progressive gastric involvement. Although subsequent further investigation will be required, magnified endoscopic observations and recognition of abnormal branched microvasculature may facilitate detecting gastric involvement in patients with follicular lymphoma.

In case 1, the gastric pits were found in an irregular pattern but were nonetheless intact. In contrast, case 2 exhibited diffuse destruction of gastric pits with only limited sections showing intact pit structure. We speculate that the structural differences between the two cases reflect distinct pathologies. The destruction of gastric pits observed by endoscopy in case 2 seems to result from the loss of epithelial cells (Figure 4(c)). On the other hand, in case 1 the gastric pits though irregular seem to have been preserved along with the epithelial cells. The presence of granulation tissue in case 2 suggests that chronic inflammation caused by follicular lymphoma cell infiltration led to damage of the gastric epithelial cells, finally resulting in the destruction of pit structure. Since our interpretation is based on the observations derived from only two cases, further investigation will be required to reveal the pathological and endoscopic features of gastric follicular lymphoma. Nonetheless, it is likely that gastric pits are not uniformly affected and may be intact or destroyed, unlike MALT lymphoma cases.

In conclusion, we treated two patients with follicular lymphoma involving the stomach. Abnormal branched microvessels were observed in both cases, whereas gastric pits were preserved in one patient and destroyed in the other. Although appropriate pathological assessment with immunohistological analysis of biopsy samples is essential for definitive diagnosis, magnified endoscopy of microvascular structures and gastric pits may be useful in alerting physicians to the potential for lymphoma lesions in the stomach.
